# Evaluation of the use of alternative sample types for mosquito-borne flavivirus surveillance: Using Usutu virus as a model

**DOI:** 10.1016/j.onehlt.2022.100456

**Published:** 2022-11-13

**Authors:** Nnomzie C. Atama, Irina V. Chestakova, Erwin de Bruin, Tijs J. van den Berg, Emmanuelle Munger, Chantal Reusken, Bas B. Oude Munnink, Henk van der Jeugd, Judith M.A. van den Brand, Marion P.G. Koopmans, Reina S. Sikkema

**Affiliations:** a Viroscience, Erasmus Medical Center, Rotterdam, the Netherlands; b Vogeltrekstation – Dutch Centre for Avian Migration and Demography, NIOO-KNAW, Wageningen, the Netherlands; c Division of Pathology, Utrecht University, Utrecht, the Netherlands; d Dutch Wildlife Health Centre (DWHC), Utrecht, the Netherlands; e Centre for Infectious Diseases Control, National Institute for Public Health and the Environment, Bilthoven, the Netherlands

**Keywords:** Usutu virus, West Nile virus, Arboviruses, Flavivirus, Wild birds, Surveillance

## Abstract

Wild birds are reservoirs of several zoonotic arboviruses including West Nile virus (WNV) and Usutu virus (USUV), and are often monitored as indicators for virus introduction and spread. To optimize the bird surveillance for arboviruses in the Netherlands and to explore the possibilities for citizen science in surveillance, we investigated the suitability of using alternative sample types from live and dead birds. The sensitivity of molecular detection via RT-PCR of viral RNA in feather, heart, lung, throat and cloaca swabs from dead birds, and serum, dried blood spots (DBS) and throat and cloaca swabs from live birds were compared. IgY antibody detection was also assessed from DBS relative to serum on protein-microarray and virus neutralization test. Feathers showed a high detection sensitivity for USUV RNA in both live and dead birds, and no significant decrease was observed in the RNA loads in the feathers after being stored dry at room temperature for 43 days. Additionally, viral RNAs extracted from feathers of day 0 and 43 were successfully sequenced. The results indicated no statistical significant difference in sensitivity and viral loads detection in heart, spleen, and lung relative to corresponding brain samples in dead birds. In live birds, viral RNA loads did not differ between throat and cloaca swabs. This study identified less-invasive sample types that allows involvement of citizens in collecting samples from wild birds for arbovirus surveillance. Sensitivity and specificity of DBS-based antibody detections were significantly lower and therefore need optimization.

## Highlights

• The study examined the use of less invasive alternative samples for flavivirus surveillance in birds.
• Feathers showed a high detection sensitivity for USUV RNA in both live and dead birds.
• USUV RNA loads detection in heart, spleen, and lung was comparable to brain samples in dead birds.

## 1 Introduction

Wild birds play a role as amplifying hosts and in the transmission cycle for several arboviruses including members of the *flavivirus* and *alphavirus* genera such as West Nile virus (WNV), Usutu virus (USUV), St. Louis encephalitis virus (SLEV) and Sindbis virus (SINV) [1,2]. These viruses cause disease in humans, livestock, and occasionally in the reservoir wild birds [3]. The bird species identified as likely amplifying hosts for these viruses, are part of different taxonomic families and may play varying roles in their introduction and spread to new or endemic areas [[3], [4], [5]]. In Western Europe, USUV and WNV are mainly detected in birds belonging to the Orders Passeriformes, Strigiformes, Falconiformes, Columbiformes, Piciformes, Apodiformes and Anseriformes [6].

USUV and WNV are both vector-borne flaviviruses of the Japanese encephalitis serogroup and are transmitted mainly by *Culex* spp.*,* but occasionally also by *Aedes* spp. of mosquitoes [7,8]. Both viruses are maintained in sylvatic circulation through a bird - mosquito transmission cycle. They also often co-circulate in locations where they are endemic [[9], [10], [11]]. USUV has caused outbreaks and mortality mainly in wild blackbirds (*Turdus merula*) and captive great grey owls (*Strix nebulosa*) across Europe and has resulted in a massive decline in blackbird population following USUV outbreaks [6,12,[13], [14]]. Humans are dead-end hosts for USUV and WNV. The first human cases of USUV neuro-invasive disease in Europe were reported in Italy in 2006 in immunocompromised patients [,[15], [16]]. Subsequently, antibodies to USUV were detected in blood donors from enzootic areas in Italy, Germany, and Austria between 2011 and 2018 [[17], [18], [19], [20]], and in the Netherlands in 2018 [21]. These findings thus emphasized the zoonotic potential of USUV [22] and the importance of strengthening surveillance for early detection of arboviruses in reservoir hosts as an early warning system. Following the detection of WNV in humans during an outbreak in 1962/63 in France [23], WNV re-emerged in Romania, Italy, Russia and France in 1996, 1998, 1999 and 2000 respectively [24]. Afterward, West Nile virus has spread widely across parts of Europe and has caused significant morbidity and mortality in horses [25] and humans [,^15^,]. In birds, however, only limited mortality has been observed within Europe [[26], [29]], unlike what was observed across Northern America [[27], [28]].

Surveillance systems for zoonotic viruses in wildlife have early detection and informing public health actions as important objectives [30]. To achieve these objectives and ensure efficient performance of a surveillance system, the sensitivity, simplicity (i.e. ease of operation and data collection), and costs are key attributes [31]. A common methodology for monitoring the introduction and spread of arboviruses having wild birds as reservoirs is collecting and screening samples such as throat swabs and blood for viral RNA and serum for antibody detection in live birds, or organs (e.g. brain) from dead birds for virus RNA detection [9,[32], [33], [34], [35], [36]]. In birds, viraemia is only observed within an average of 5–7 days post-infection with WNV [37] or USUV [38]. Therefore detecting a single infection through RT-PCR often requires a large number of birds to be sampled and tested and thus there is an increased likelihood of underestimating the true disease prevalence. The use of serology in surveillance to detect antibodies (IgM or IgG) provides an opportunity to infer more accurately the extent of temporal and spatial exposures in the population of interest [39]. Serum samples are often tested for antibody detection, but it is challenging to collect adequate volumes from the potential reservoirs, which are often small passerine birds. Another challenge in serology for WNV and USUV is the immunologic cross-reactivity among flaviviruses, which complicates interpretation of serological tests especially in situations where there is co-circulation of viruses within the same antigenic group, thereby exposures are often only distinguished through an approach that includes multiplex testing, virus neutralization testing and taking an extensive medical history including travel and vaccination history [40].

Identifying sample types that are easier to collect and less invasive to the host, while also being sensitive for virus and viral load detection could aid in achieving the surveillance objectives. To optimize bird surveillance systems for early detection and monitoring of USUV and WNV presence, we examined the suitability of different alternative sample types from live and dead birds for virus or antibody detection. We compared the sensitivity and viral loads in samples that are less laborious to collect during necropsy (i.e. heart, spleen, and lungs), and those which are less invasive on the host (i.e. feather) to the conventionally used samples (i.e. brain and throat swabs) for dead and live birds respectively. Utility of dried blood collected on protein saver cards (DBS) was also assessed relative to conventionally used serum in live birds for antibody detection.

## 2 Materials and methods

### 2.1 Sample collection

#### 2.1.1 Live and dead bird surveillance

Samples used in this study were collected between 2016 and 2021 as part of a wider study on the presence of arboviruses in birds in the Netherlands (performed under the ethical permit AVD801002015342 issued to NIOO_KNAW). Two strategies were used to collect samples: 1. from healthy wild birds that were captured during regular monitoring surveys and 2. from found dead birds that were submitted to the Dutch Wildlife Health Centre (DWHC) through citizen science efforts. In the live bird monitoring, samples collected included dried blood spots (collected on Whatman 903 protein saver cards; Whatman 903™, GE Healthcare, USA), feathers, throat swabs, cloacal swabs and serum (blood from live birds were spun down at 10,000 x*g* for 5 min to collect serum). The throat and cloacal swabs were collected separately in 2016, but pooled in a single vial with 1.2 mL of virus transport medium (VTM) for each bird sampled between the years 2017–2021. In the dead bird monitoring scheme, samples collected from dead birds included brain, heart, lung, feathers, throat swabs and cloacal swabs. Feather samples from live birds were collected only between 2019 and 2021 and stored dry. All samples were stored at -80 °C until use except the dried blood collected on protein saver card (DBS) which were stored at -20 °C.

#### 2.1.2 Sensitivity of feathers for USUV detection after storage at room temperature

**Fig. 1:**
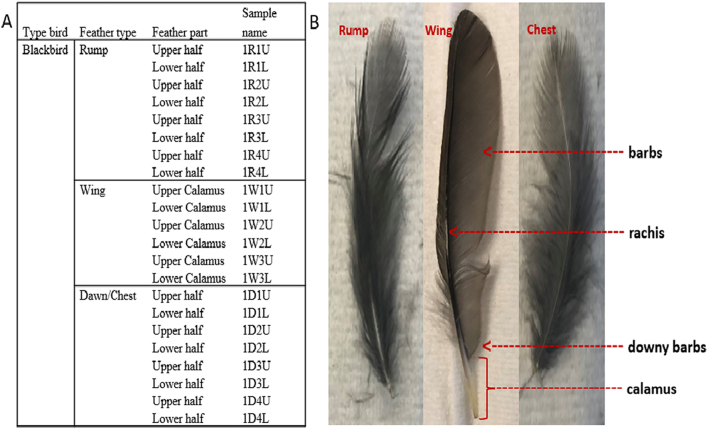
Summary of the molecular testing strategy for upper and lower calami of three feather types (wing, chest, and rump). A. Different samples were taken from the feathers: 4×, 3×, and 4× each of upper and lower calami of rump, wing, and chest feathers were tested at each time-point (day 0, 22, and 43). B. Sample of each feather type collected per dead bird and each calamus was halved into upper and lower parts for testing.

To experimentally determine the suitability of using feathers for virus detection in the field, feathers were collected from 10 dead blackbirds (*Turdus merula*) that tested positive for USUV RNA via RT-PCR on brain samples. Rump, wing, and chest feathers were detached from each bird to determine the suitability of using different feather types for detecting USUV RNA after detachment from a bird, and after storage at room temperature for 22, and 43 days. The shaft of each rump or chest feather was cut in half. These were further split in half vertically. For the wing feather, the calamus was halved and divided into the upper and lower parts relative to the proximal umbilicus (Fig. 1 B). Eleven feathers in total (4× rump, 4× chest, and 3× wing) were processed per bird, yielding 22 samples per bird per timepoint (Fig. 1).

### 2.2 Sample preparation

#### 2.2.1 Swabs and organs

Brain samples were first tested via RT-PCR, whereas the other sample types were tested only when brain samples were USUV RNA positive, except for samples collected in 2016 where all sample types were concurrently tested. A half pea size of tissue was homogenized in lysis buffer using the Fastprep bead beater (brain at; 4 m/s for 20 s, and heart, liver, spleen, lung, and kidney at; 5 m/s for 30 s). Samples were subsequently centrifuged at 10,000 x*g* for 5 min and nucleic acid (NA) was extracted from the supernatant using the Roche MagNA Pure system with Phocine distemper virus (PDV) as internal NA extraction control [41]. 600 μL of swab material in viral transport medium (VTM) was eluted in 600 μL external lysis buffer and NA extracted using the Roche MagNA Pure with PDV as an internal control.

#### 2.2.2 Dried blood spots

For molecular detection, each dried blood spot sample (DBS) collected on Protein saver cards (Whatman 903™, GE Healthcare, USA) was processed by cutting out 5 disks (∼100 μL sample) - using a 6 mm diameter hole punch (Uxcell®) - from a fully saturated part of the card and eluting in 300 μL lysis buffer. The sample in lysis buffer was incubated for 2 h (shaking; 400 rpm) at room temperature and 200 μL DMEM medium was added and centrifuged for 2 min at 1500 *g*. Furthermore, NA was extracted from 50 μL of supernatant using the MagNA Pure LC instrument ™(Roche) with the Total nucleic acid extraction kit according to the manufacturer's instructions. For antibody testing, one puncture of 3 mm diameter of fully saturated DBS (equivalent to 1.5 μL serum) was cut out from the protein saver card and eluted in 40uL Phosphate buffered saline (PBS) and Tween-20 Surfact Amps Detergent Solution (Thermo Scientific, *Rockford*, *IL*, *USA*).

#### 2.2.3 Feathers

Half of the calamus of each included feather was homogenized separately in tissue lysis buffer with ceramic beads, using a Magnalizer (60 s; 6500 rpm) as previously described but with minor adaptations [42]. Samples were spun down at 10,000 x*g* for 3 min, and NA was extracted as done for the swabs and tissues from organs. NA was extracted from 4×, 4×, and 3× of the lower and upper calamus of each of the rump, wing, and chest feathers of individual birds on day 0, and repeated using the same protocol on days 22 and 43. The average values of the viral loads (log_10_ copies/μL) from the different sections of each calamus (upper or lower) were finally used for comparative analyses.

### 2.3 USUV and WNV RNA detection

All NA extracted from live and dead bird samples were screened for the presence of USUV and WNV using real-time PCR with primers and probes as previously described [43,44] (Table 1). The positive results from samples tested were further confirmed by a second USUV or WNV confirmation PCR targeting different loci of the viral genome [[45], [46]]. Samples were considered positive when the Ct value was <40 in both PCRs.

**Table 1:** Primer- and probe sequences used for USUV and WNV screening.

Target	Forward primer (5′ – 3′)	Reverse primer (5′ – 3′)	Probe (5′ – 3′)
USUV NS5	CAAAGCTGGACAGACATCCCTTAC	CGTAGATGTTTTCAGCCCACGT	AAGACATATGGTGTGGAAGCCTGATAGGCA
WNV C	CCACCGGAAGTTGAGTAGACG	TTTGGTCACCCAGTCCTCCT	TGCTGCTGCCTGCGGCTCAACCC
PDV HA	CGGGTGCCTTTTACAAGAAC	TTCTTTCCTCAACCTCGTCC	ATGCAAGGGCCAATT

### 2.4 Antibody detection

#### 2.4.1 Serum

All bird sera were screened using the protein micro-array technique as previously described for USUV and WNV antibody detection in horses [47]. The serum samples were tested on 64 pad nitrocellulose glass plates coated with duplicate spots each of USUV and WNV purified NS1 antigens (USUV, The Native Antigen Company, Kidlington UK; WNV, Sino biologicals, China). Slides were incubated with sera and subsequently with Alexaflour-647 conjugated goat anti-duck IgY 647 (*Jackson Immunoresearch Laboratories*, Inc., West Grove, USA) at 1:500 dilution. Tested slides were scanned with a Tecan Power Scanner (Tecan Trading AG, Mannedorf, Switzerland) and the median fluorescence signals quantified as previously described [48]. Sera were tested on a 1:80 dilution for IgY (an equivalent of IgG in mammals) reactivity and samples with a signal above background (≥ 6000) were selected for confirmation on a virus neutralization test (VNT) as previously described [48]. In summary, the VNT was performed using titrated stocks of USUV (GenBank: MN122148.1) and WNV (GenBank: AY532665.1). The serum was heat-inactivated at 56 °C 30 min, followed by incubation of two-fold dilution series of serum with 100TCID50 of the respective virus cultures and transfer to Vero cells (Vero ATCC CCL-81). Five days post-inoculation (dpi), viral cytopathic effects (CPE) were recorded post-inoculation for USUV [47,48], and at 7 days post-inoculation for WNV. Included in each experiment were positive and negative controls, virus back titration, and serum only controls. The reciprocal of the highest serum dilution at which virus replication was fully blocked was set as the VNT titre. Samples were tested with a starting dilution of 1:8 due to the limited volume of serum. Sera were defined as positive when a minimum of two-serial dilutions showed complete blocking of viral infection (cut-off titre ≥16). A serum was defined as confirmed-positive for USUV antibodies only when reactivity for USUV on the protein micro-array was confirmed by comparative VNT for USUV and WNV, with a titre for USUV at least 4-fold higher than a WNV titre on the VNT. Alternatively, a serum was considered WNV positive when confirmation by WNV VNT gave a 4-fold higher titre relative to the USUV VNT and titre ≥16.

#### 2.4.2 Dried blood spots

Dried blood spot (DBS) samples were also tested by protein microarray at 1:80 dilution on 94 pad nitrocellulose glass plates coated with NS1 antigens of flaviviruses as done with the sera samples above, after elution in 40 μL PBS. No VNT confirmation of detections from the protein microarray was performed for the DBS samples as there was only little volume of blood left on the DBS cards and confirmation on the VNT would require more volume. Hence, fluorescence signals from the DBS on the PMA were compared with VNT results from sera, which is considered the gold standard confirmation test.

#### 2.4.3 Sequencing of viral genomes from feathers

RNA samples from 14 positive feathers of confirmed positive blackbirds were selected for sequencing. These included 9 RNA samples which were extracted from feather calami at day 0 and 5 RNA samples extracted from feather calami at day 43 after storage at room temperature. Selection among samples from Day 0 and Day 43 was based on them having a C_T_ value below 32. USUV genomes were generated using a specific USUV multiplex PCR on the Oxford Nanopore MinION platform as previously described [49]. The libraries were generated using a Native Barcoding Kit from Oxford Nanopore Technologies (SQK-LSK109) and sequenced on an R9.4 flow cell.

#### 2.4.4 Sequence data analysis

Raw sequence data were demultiplexed using Porechop (https://github.com/rrwick/Porechop). A reference-based alignment was performed using Geneious. The consensus genome was extracted and positions with <30 were replaced with an ‘N'. Homopolymeric and primer binding regions were manually checked and resolved by consulting previously obtained reference genomes.

### 2.5 Statistical analysis

Viral loads (in RNA copies/μL of sample) were estimated from C_T_ values of real-time PCRs using the standard curve method in an LC480 software® based on a 10-fold serial dilution of a cultured WNV or USUV that is used as a positive control [50]. We calculated the sensitivity (detection probability) for virus detection in each sample type with an associated Wilson score 95% confidence interval for binomial proportions.

Relative sensitivity and viral loads (RNA copies/μL) detected across sample types were compared from a subset of dead and live birds that had tested positive for USUV on brain samples or throat swabs respectively, and for which other paired specimen types were also tested.

Correlation between viral loads in the brain and other sample types (heart, spleen, lung, cloaca swab, oropharyngeal swab and feathers), and between swabs or serum and DBS were estimated using the Spearman correlation test. The normality of data was checked using Shapiro-Wilk test. For normally distributed data, viral loads (log_10_ RNA copies/μL) were compared between or across sample types using *t*-tests or analysis of variance (ANOVA) respectively. Otherwise, a non-parametric test equivalent (Wilcoxon sign ranked test with continuity correction or Kruskal-Wallis test) was used. Sensitivities and specificities of DBS and serum on the protein microarray were estimated using ROC-curve and titres from protein microarray data were estimated using GraphPad prism (v9.3.1). All other analyses were done in R software (v4.1.2).

## 3 Results

In the dead bird monitoring, samples from 1304 dead birds were tested for USUV and WNV RNA within the study period, and a subset of 64 USUV brain-positive birds that had other paired samples tested were selected for the analyses (S1). For the live bird surveillance, 28,169 birds were tested and 64 USUV swab positives and 4 WNV swab positive birds were selected for analyses in this study. 15 of the 64 USUV positives had both throat and cloaca swabs tested and 47 swabs (throat, cloaca or pooled) had paired DBS samples tested for USUV RNA. A subset of 58 serum samples that had paired DBS samples were tested on both PMA and VNT (for sera only) (S1).

### 3.1 Invasive versus less-invasive sample types in dead birds (USUV)

**Fig. 2:**
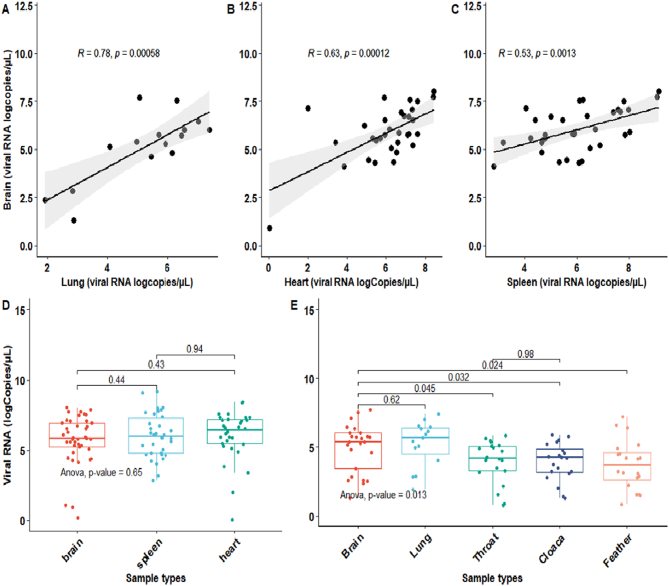
Comparison of USUV viral RNA loads (log_10_ RNA copies/μL) across different specimen types of dead birds*.* A, B & C. Correlation of USUV viral RNA loads in different sample types from dead birds (Spearman's correlation). D & E. Variability in viral loads across different sample types relative to brain samples from dead birds (student t-test and analysis of variance).

Samples from 64 USUV positive dead birds, mainly passerines, were included in the comparative analyses (S2). The USUV detection sensitivities in organ samples (lung, heart and spleen) were between 91 and 95% when compared to the paired brain samples, with the highest sensitivity in the lung tissue (95%) (S3). In comparison to brain samples, the less invasive samples (feather calamus, throat swab, and cloaca swab) had sensitivities between 88 and 92% with the highest sensitivity detected in the feather calami (92%) (S3). There was no significant difference in viral RNA loads (log_10_ RNA copies) detected in the positive organs relative to the brain samples (Fig. 2 D). The combined median viral loads in the less invasive sample types (swabs and feathers) did not differ significantly from that in organ samples, although the less invasive samples had slightly lower median viral RNA loads (Median 0.88 [95%CI 0.78, 0.94] log_10_ RNA copies/μL versus Median 0.93 [95%CI 0.86, 0.97] log_10_ RNA copies/μL).

### 3.2 Sensitivity of feathers for molecular detection of USUV

**Fig. 3:**
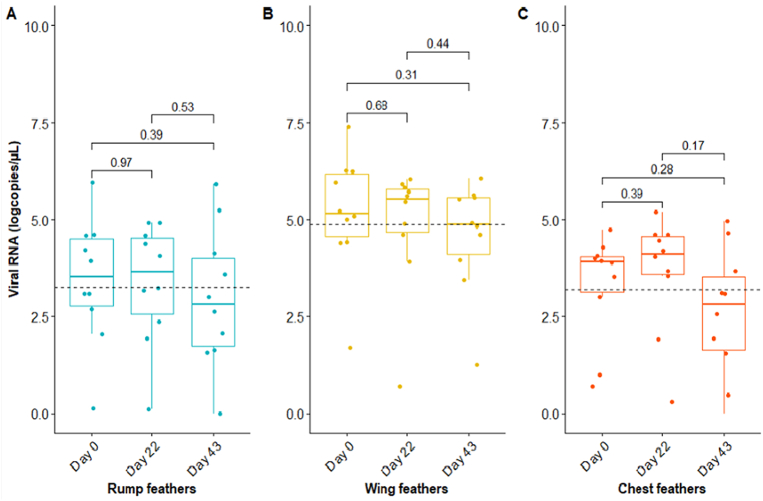
USUV RNA detection levels in feathers of dead birds over storage time*.* A B & C shows the comparison of viral loads within each feather type across time points (Day 0, 22 & 43). Log_10_ RNA copies/μL of samples were compared across each other using analysis of variance (ANOVA).

The possible use of feathers in surveillance was further investigated by comparing feather types, feather elements (upper and lower calamus) and storage duration. The viral loads (copies/μL of sample) in the upper and lower calami of each of the three feather types (rump, wing and chest) collected from 10 dead Eurasian blackbirds were determined via RT- PCR. The lower calamus of all feather types yielded significantly higher viral loads than the upper calamus for wing, chest and rump feathers, with highest loads in wing feathers (paired *t*-test; *p* = 0.02, 0.02 and 0.004 respectively) (S3). No difference was detected between the lower calami of rump and chest feathers. USUV RNA was still detected in all three feather types after storage at room temperature for 22 and 43 days. Across all feather types, there was only a minor decrease in the median RNA load between day 0 (median 4.06 log10 RNA copies/μL) and 43 (median 3.59 log10 RNA copies/μL), but with no significant difference between the time-points (Fig. 3). Furthermore, USUV near complete-genome sequences could be generated from all 14 RNA samples from the feathers of days 0 and 43 that were submitted to sequencing. All obtained sequences covered >90% of the USUV genome. Sequences obtained from feathers of different individuals were not identical and all belonged to the lineage Africa 3. The sequences were closely related to other USUV genome sequences obtained from blackbirds in the Netherlands. For feathers tested and sequenced at days 0 and 43, sequences obtained at the two timepoints were identical. The USUV genome sequences from this study have been deposited in the GenBank database under the accession numbers ON755209 to ON755222.

### 3.3 Sample types for live bird surveillance (USUV)

Sixty-five USUV positive live birds were included in the live bird sample analysis. Of these samples, 15 USUV positive blackbirds collected in 2016 had pair-matched cloaca and oropharyngeal swabs taken and their viral loads compared. There was no significant difference in quantities of viral RNA between the throat and cloaca swabs (paired *t*-test; *p* = 0.28).

Of forty-seven USUV swab-positive (throat, cloaca or pooled throat and cloaca) live birds, corresponding DBS samples were also tested and compared for sensitivity and difference in USUV viral loads. The DBS samples had a lower sensitivity (53% detection) compared to the swabs with a statistically significant difference (Wilcoxon sign-rank test; *p* < 0.001, *r* = 0.48, 95% CI[0.22, 0.68]). Excluding birds with negative DBS results, the viral loads in DBS was not significantly different from pooled swabs (Wilcoxon sign-rank test; *p* = 0.299, *r* = 0.17, 95% CI[0.15, 0.46]) (s4 A). To further study the possible use of DBS for USUV detection, we randomly selected and tested 94 DBS samples from swab negative birds that were sampled within the same periods and locations where USUV was detected (2016–2020.) One of the swab-negative samples tested positive for USUV on DBS (1/94; 1.0%) and had a viral load of 1.17 log_10_ RNA copies/μL.

### 3.4 West Nile virus in live birds

West Nile virus was detected for the first time in the Netherlands in the summer of 2020 through the live bird surveillance scheme in 4 individual live birds [46]. No WNV positive dead birds were found. The live birds tested positive for WNV via pooled throat-cloaca swabs and had viral loads ranging from 2.08 to 5.77 log_10_ viral RNA copies/μL (median 2.72 log_10_ RNA copies/μL). From four swab positive birds (song thrush, house sparrow, great tit and a chicken), faecal samples were available and tested. From the positive chicken, a feather was also collected. The swab-positive song thrush (3.20 log_10_ RNA copies/μL) was WNV positive on both faeces (3.76 log_10_ RNA copies/μL) and feather (3.04 log_10_ RNA copies/μL) with similar viral loads. Faecal samples from the other two passerine species and chicken tested negative. A WNV near-complete genome sequence was generated from RNA of the faecal sample and the genome sequence has been deposited in GenBank with accession number ON755223. The other 3 faecal samples from swab-positive birds were negative for WNV.

### 3.5 Dried blood spots as an alternative for serum collection

**Fig. 4:**
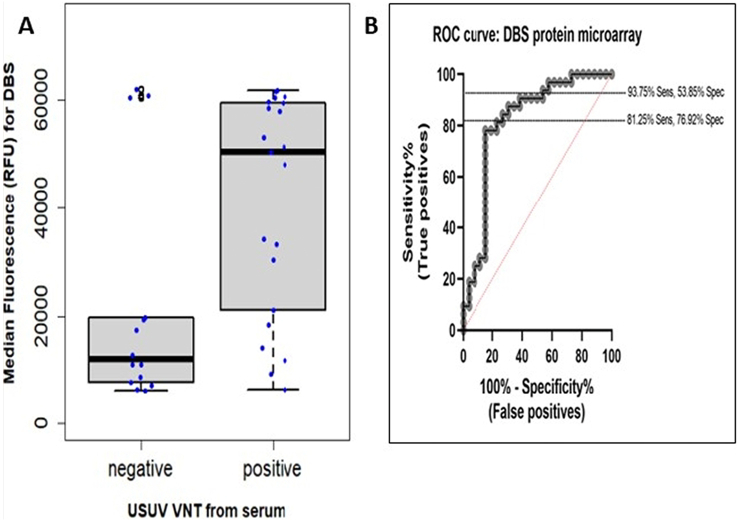
DBS in serology for USUV IgY antibody detection in live birds. A. Relationship between median relative fluorescence unit (RFU) values of DBS on the protein microarray to confirmed VNT results from serum testing (*n* = 35). B. ROC curve of median RFU values for DBS on the protein microarray estimated relaitve to corresponding VNT- confirmed serum samples.

Paired DBS and serum samples were collected from 52 common blackbirds (*Turdus merula*) and 6 song thrushes (*Turdus philomelus*). Antibody binding to the USUV NS1 antigen was measured by protein microarray (PMA). We assessed the correlation between the median fluorescence values obtained from dried blood spots (DBS) on the PMA and median fluorescence values from corresponding serum samples (Y-axis), using Spearman correlation. The DBS had a low positive correlation to serum (Spearman correlation: *R* = 0.69, 95%CI [0.48, 0.84]). (. A confirmatory virus neutralization test (VNT) could only be performed on the serum samples. The USUV VNT confirmatory titres ranged from 1:16 to ≥1:1024. Overall titres from sera on the USUV VNT ranged from 1:4 to 1:1536, whereas on the WNV VNT titres ranged between 1:4 and 1:32. Using VNT-positive sera as the golden standard, a ROC curve was plotted for the protein array results of the DBS. The most optimal derived sensitivity and specificity of the DBS relative to corresponding VNT-confirmed sera were 81.25% and 76.92% respectively (Fig. 4 A & B).

## 4 Discussion

In this study, we examined the use of less invasive samples from both dead and live birds for molecular and serological detection of USUV and WNV during wild bird surveillance. In dead birds, we showed that feathers and other less invasive samples such as throat swabs and cloaca swabs can function as alternatives for brain samples in dead bird surveillance for USUV. In live birds, the use of feathers can also potentially substitute throat swabs, which would greatly simplify sample collection. Moreover, the limited effect of storage at room temperature on detection sensitivity may indicate that feather collection from the field instead of directly from live birds, may be considered.

In our study, feathers showed a high sensitivity for USUV RNA detection which was not significantly different from paired brain samples, although the median viral load in the feathers was significantly lower. We also detected WNV in the feather (1/1) of a live bird (Song thrush, *Turdus philomeus*), thus further indicating that feathers could also be sensitive for the detection of other flaviviruses in birds. In addition, we show that it is possible to detect USUV RNA from feathers until 43 days. Although we did not test moulted feathers, future experiments should focus on testing the suitability of feathers from moulting birds, random field feather samples or archived feathers from museum samples to support surveillance and possibly address issues with predicting the time of pathogen introduction by testing retrospectively. The possibility of using feathers collected from bird roosts makes it a valuable tool for sampling. For instance, raptors are good sentinel species for monitoring viral circulation due to their suspected risk of infection via preying on infected small reservoir birds, but are difficult to trap and sample using conventional methods [51]. Asides from collecting feathers from bird roosts, feathers can replace the collection of dead birds which often requires cold chain storage which is most of the time impractical in the field.

All tested tissues (lungs, heart, and spleen) from the organs of dead birds showed high sensitivity and yielded good viral loads which were not significantly different from paired brain samples. In addition to offering good detection, these organs are easier and less laborious to collect than brain samples, and present a lower risk to the pathologist during necropsy. Also in dead birds, throat and cloaca swabs had a similar sensitivity as paired brain samples, although they had lower viral loads. In other studies with throat and cloacal swabs, low viral loads were generally observed and inconsistent results were found across species [[52], [53], [54]]. So, throat and cloacal swabs can serve as relevant tools for monitoring the prevalence and evolution of USUV in both live and dead birds. Similarly, as with feathers, throat and cloaca swabs can also be collected from dead birds as an alternative to submitting whole dead birds, and when performing a necropsy presents some risk or is impractical.

Following our investigation of the sensitivity of DBS on the protein microarray as a seroepidemiology tool, the DBS had a similar sensitivity as corresponding serum but had relatively low specificity. In flavivirus serology, virus neutralization assays are the golden standard, due to cross-reactivity of antibodies between flaviviruses. Virus neutralization assays have been set up for use in DBS, but due to the low volumes of collected blood this could not be done in this study. Therefore, the use of DBS for antibody detection is currently not suitable for individual diagnostics. The high sensitivity of antibody testing using DBS with the PMA could make it suitable for monitoring trends of known enzootic flaviviruses or as a screening tool. However, in the case of co-circulation of related flaviviruses, as is the case for USUV and WNV, setting up a specific conformational test would be essential [55,56].

To conclude, we identified alternative samples that could be used for surveillance of arboviruses in live birds, and we described for the first time the sensitivity of feathers for USUV RNA detection in passerine birds. However, a small sample size was used for most comparisons and the species included were dominated mostly by Eurasian Blackbirds as there were only a minimal number of other positive species, therefore we were unable to estimate variability across species. The fact that samples were not weighed before testing may also limit our conclusions. However, the ability to detect viral RNA in samples like feathers and faeces provides a great advantage for sample collection through citizen science. Although there were only a few faecal samples tested, further studies would be necessary to evaluate their sensitivity and use in bird surveillance for flaviviruses. Collecting post-mortem throat and cloacal swabs from dead birds can avoid the tedious steps and risks involved in obtaining tissues under field conditions. Finally, due to the transient viraemic period in WNV and USUV infected birds, a combination of molecular and serological screening of samples would increase the sensitivity of the surveillance system. However, collecting sufficient volume of sera from small passerine birds for serologic screening without harming the birds is often impossible. Therefore, the use of DBS may serve as a good alternative to monitor the extent of arboviral exposures through antibody detection, although further development of conformational testing is necessary when related flaviviruses co-circulate.

## Authors' contribution

RS conceptualized the study. NA, IC, & EB contributed to molecular and serological screening of samples. NA analysed the data and wrote the first draft of the manuscript. RS, EB, TB, EM, CR, BM, HJ, JB, & MK significantly contributed to reviewing and editing. All authors read and made significant inputs and agreed on the last version of the paper.

## Funding

This work is part of a One Health PACT the research programme with project number 109986, which is (partly) funded by the Dutch Research Council (NWO). RS and HJ received funding from the Dutch Academy Institutes Fund 2021 (Linking ecological analysis to plant and animal historical collections for tracking urban evolution). RS and MK received funding from the European Union's Horizon 2020 research and innovation programme under grant agreement No. 874735 (WVO project).

## Conflict of interest

None declared.

## Data Availability

Data will be made available on request.
